# Supplementary material for: Selective suppression of antisense transcription by Set2-mediated H3K36 methylation
Source: Nat Commun. 2016 Nov 28;7:13610. doi: 10.1038/ncomms13610 (PMC5133703; doi:10.1038/ncomms13610)
Supplement: Supplementary Information — Supplementary Figures 1-9. [file ncomms13610-s1.pdf]

Supplementary Figure:

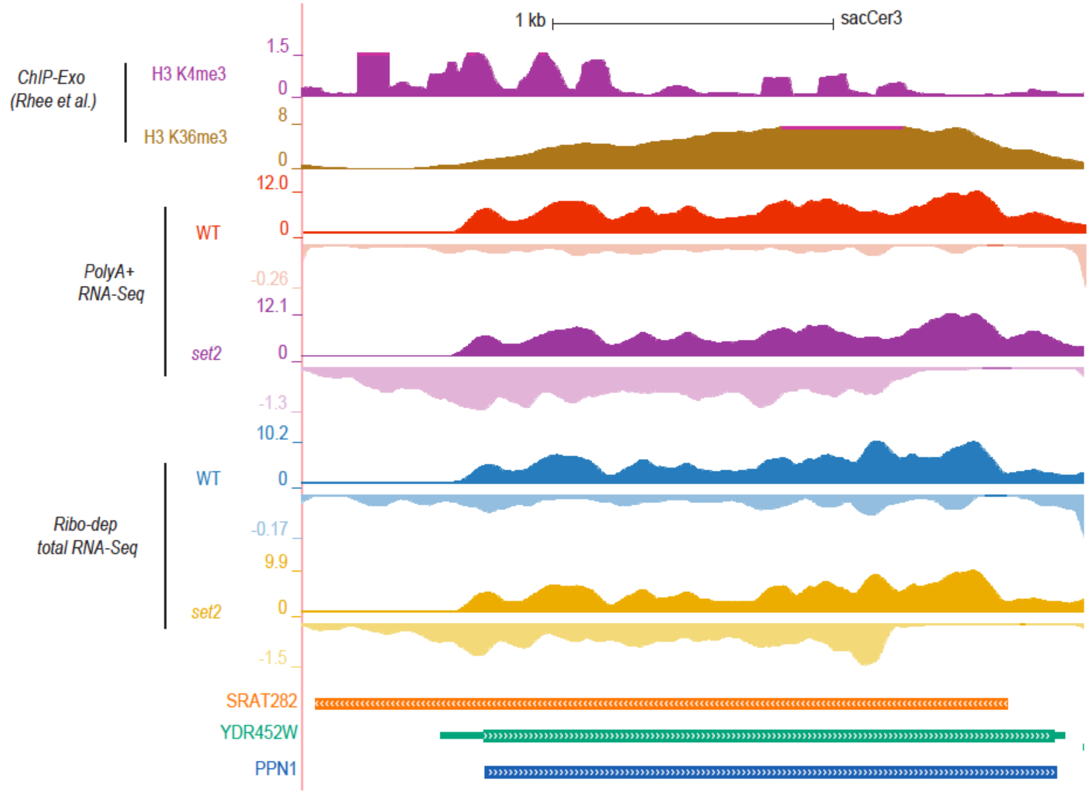

Supplementary Figure. 1: SRAT282 is transcribed at low levels in the wild type strain: Genome browser profile showing the distribution of modifications and the transcripts (both ribo-depleted and Poly A+ RNA) produced in wild-type strain (WT) and the SET2 deletion mutant (*set2*) over the gene *YDR452W*. The modifications are re-analyzed tracks from [1]. One replicate for each ChIP-Exo track of H3K4me3 and H3K36me3 distributions are shown here. Each track is separated into the sense strand (S) on top, running from left to right and the antisense strand (AS) in the bottom running from right to left. In contrast to Figure. 1A, the y-axes of the WT samples have adjusted independent of the *set2* samples, to allow visualization of the transcript.

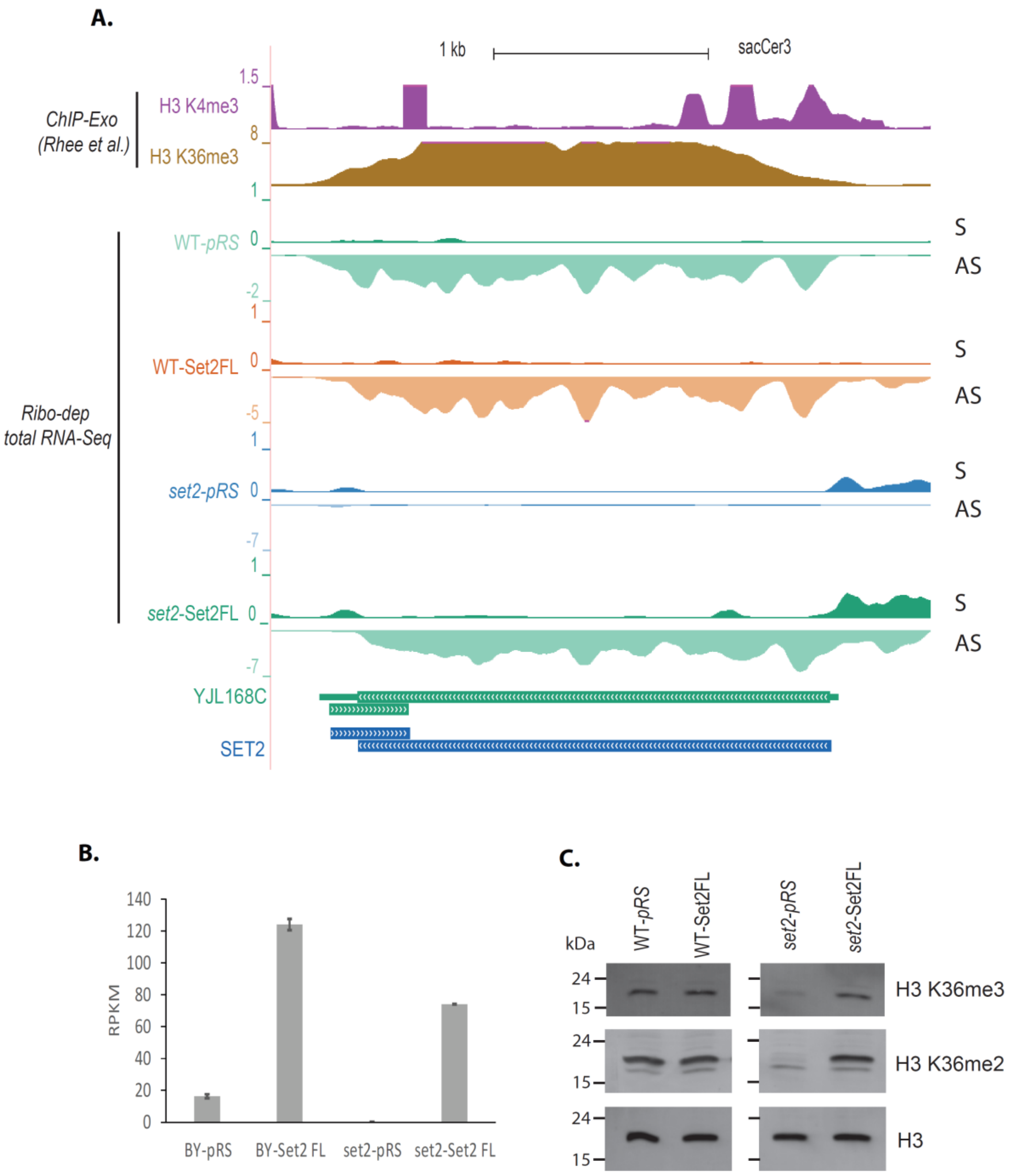

Supplementary Figure. 2: Overexpression of Set2 in the wild type BY4741 and *SET2* deletion strains: A: Genome browser profile showing the distribution of modifications and the transcripts (ribo-depleted) produced in the wild type (WT) or *SET2* deletion (*set2*) strains either with the empty vector (pRS416, denoted as pRS) or the full-length *SET2* gene (Set2FL) over the gene *YJL168C- SET2*. The modifications are re-analyzed tracks from [1]. One replicate for each ChIP-Exo track of H3K4me3 and H3K36me3 distributions are shown here. Each track is separated into the antisense strand on top, running from left to right and the sense strand in the bottom running from right to left. Both the WT samples produce the Set2 FL transcript, as does the *set2* mutant with the plasmid expressing full

length Set2. The *set2* mutant with the empty vector does not produce the transcript. **B:** Histogram of the levels of *SET2* transcript abundance in the different strains as enumerated in A above, expressed as the average RPKM from two replicates each. Set2FL is overexpressed upto 6-fold in the WT strain, and 4-fold in the *set2* mutant. **C.** Immunoblot of whole cell extracts from the indicated strains with the either H3, H3K36me2 or H3K36me3 antibodies. Results denote that despite the over expression of Set2, the levels of H3 K36 methylation remain comparable to the WT strain.

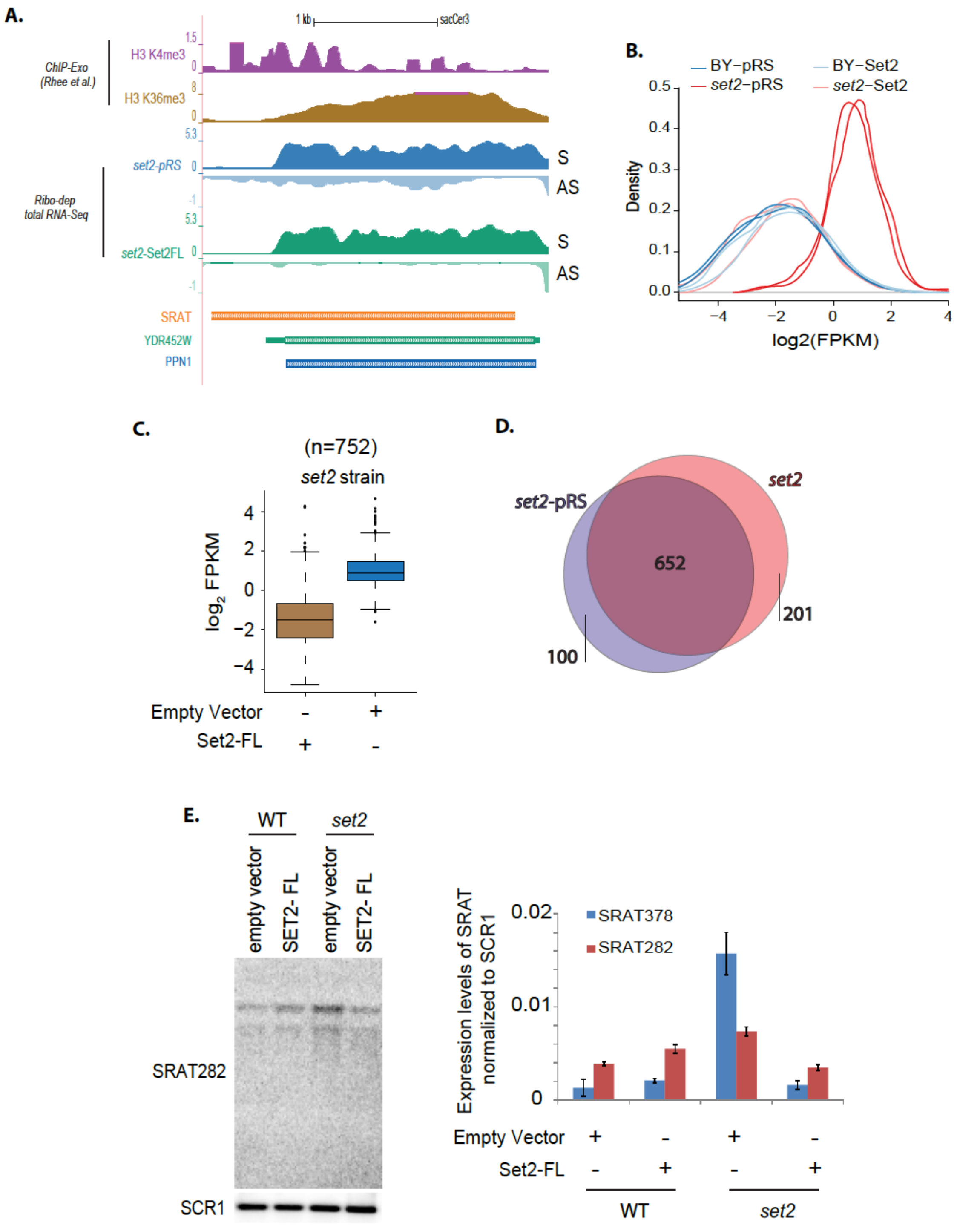

Supplementary Figure. 3: SRAT suppression depends on functional Set2: A. Genome browser profile showing the distribution of modifications and the ribo-depleted transcripts produced in the *SET2* deletion mutant (*set2*) either with the empty vector (pRS416, denoted as pRS) or the full-length *SET2* gene (Set2FL) over the gene *YDR452W*. The modifications are re-analyzed tracks from [1]. Each track is separated into the sense strand (S) on top, running from left to right and the antisense strand (AS) in the bottom

running from right to left. The expression of *SET2* full-length in the SET2 deletion strain suppresses SRAT282. **B.** Density traces of the SRAT abundance (Log2 FPKM) in the wildtype (BY) and *SET2* deletion (*set2*) strains transformed with either the empty vector (pRS) or the Set2 expression vector (Set2). Two traces of each color denote the two replicates used for RNA seq. **C.** Boxplot showing the abundance of SRATs (log2 FPKM) in the *SET2* deletion mutant either with the empty vector (pRS416) or the full-length *SET2* gene (Set2FL). The total number of genes used in this analysis is denoted above. **D.** Venn diagram showing the overlap of statistically significant SRATs produced upon deletion of *SET2* (*set2*) with those in a *SET2* deletion mutant with the empty vector (*set2-pRS*). **E.** (Left) Strand specific northern blot probing for SRAT282 using either total RNA in wild-type (WT) and *SET2* deletion mutant (*SET2Δ*) either with the empty vector (pRS416) or the full-length *SET2* gene (Set2FL). *SCR1* is used as a loading control. (Right) Quantitation of strand specific northern blots indicating the expression level of selected SRATs, normalized to the level of *SCR1* in total RNA under the indicated conditions. Error bars denote the standard error of three independent repeats.

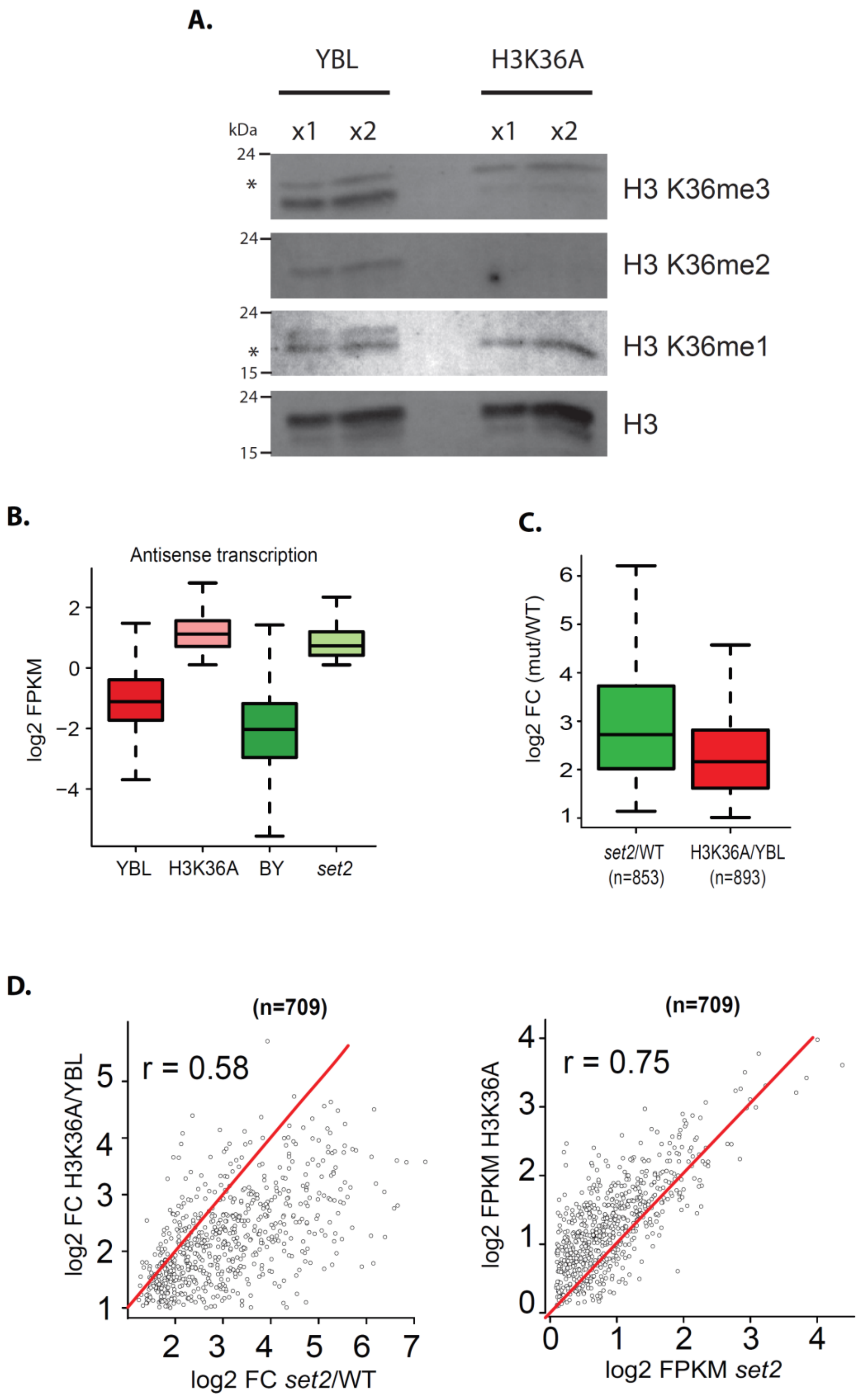
A.
YBL
H3K36A
kDa
x1
x2
x1
x2
24
*
H3 K36me3
24
H3 K36me2
24
*
15
H3 K36me1
24
15
H3
B.
Antisense transcription
log2 FPKM
YBL
H3K36A
BY
set2
C.
log2 FC (mut/WT)
set2/WT
(n=853)
H3K36A/YBL
(n=893)
D.
(n=709)
r = 0.58
log2 FC H3K36A/YBL
log2 FC set2/WT
(n=709)
r = 0.75
log2 FPKM H3K36A
log2 FPKM set2

Supplementary Figure. 4: The wild type histone shuffle strain shows a mild cryptic transcript phenotype. A. H3 K36A point mutant lacks H3 K36 methylation. Immunoblot of whole cell extracts from the indicated strains with the either H3, H3K36me1, H3K36me2 or H3K36me3 antibodies. The asterisks (*) next to selected bands in the western blots indicates non-specific bands. **B.** Boxplot comparing the abundance of SRATs (log2 FPKM) in the WT (YBL), H3K36A mutant, wildtype (BY) and Set2 deletion (*set2*) mutants. **C.** Boxplot showing the fold-change of SRAT expression (log2 FC mut/WT) in the *SET2* deletion and H3K36A mutants in comparison with their respective wildtype strains. **D.** Scatter plots either denoting the fold change in gene expression (left) or the transcript abundance (right) of 709 SRATs identified in the H3 K36A mutant. The respective values from the *SET2* deletion mutant are distributed on the x-axis, while those from the H3K36A mutant are distributed on the y-axis. The red line denotes the values where x=y. The Spearman co-efficient of correlation is provided.

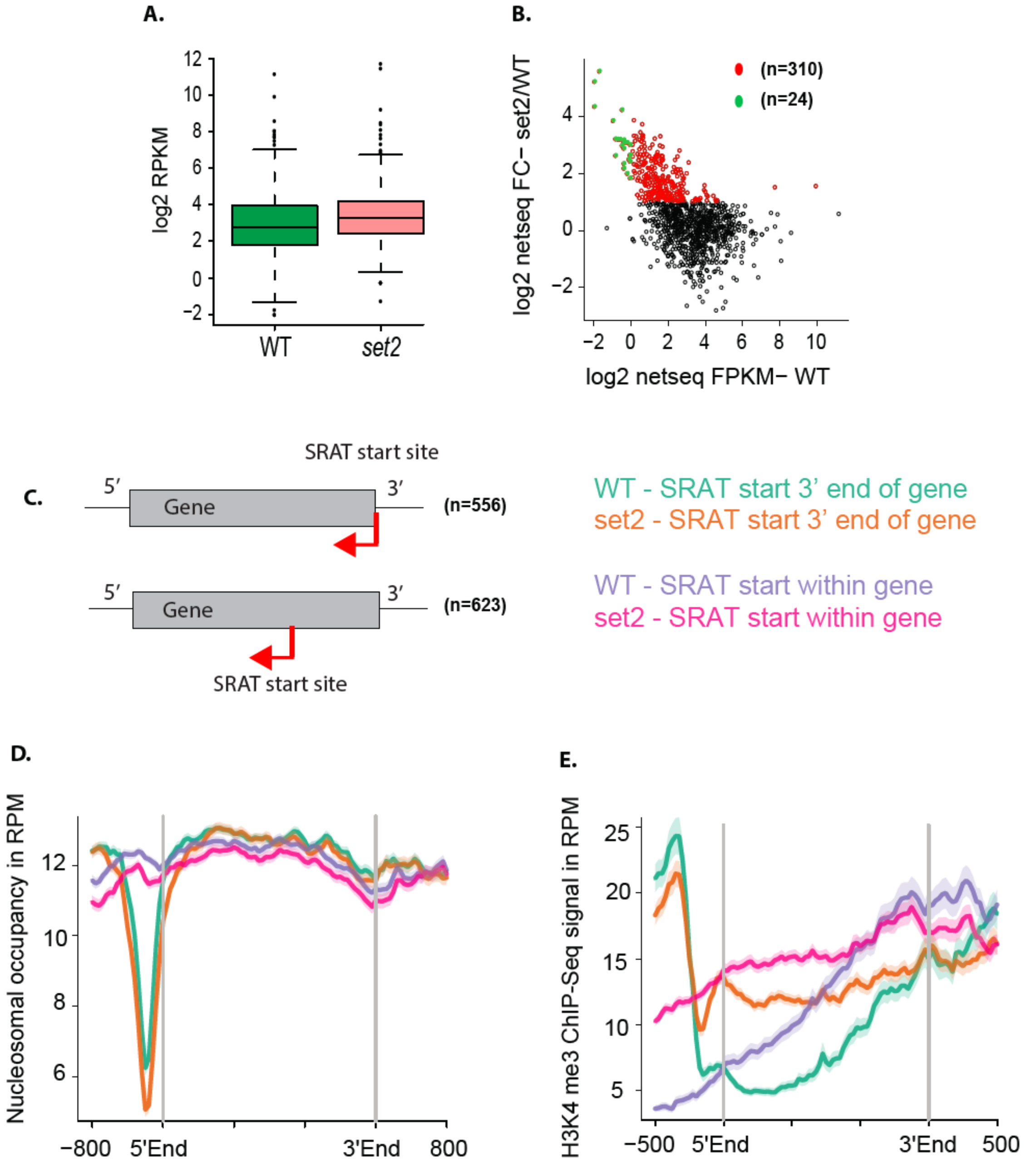

Supplementary Figure. 5: Characteristics of SRAT promoters. A. Boxplots showing the abundance of NET-Seq signal on the sense strand for 500 bp upstream of the SRAT start site, produced in either the wild-type or *SET2* deletion yeast strain. **B.** Scatter plot with the abundance of NET-Seq signal on the sense strand for 500 bp upstream of the SRAT start site in the wildtype in the x-axis and the fold change of NET-Seq signal in the *SET2* deletion mutant in the y-axis. Values that increase by 2-fold in the *set2* mutant versus the wild-type are indicated in red. Of these spots the ones with low NET-Seq signal in the WT strain are marked green. The numbers of each of these subsets are indicated in the top right. **C.** Color key for supplementary figure 5 D and E. The schematic indicates the start site of SRATs with respect to the genomic localization of the protein coding gene. SRAT

are separated according to the distance of their start sites to the 3' end of the sense genes they are embedded within. Start sites within 2 nucleosome lengths (300 bp) of the 3' end of genes are separated from those that are more than 300 bp away from the 3' end of genes. The numbers are indicated alongside the schematic. **D.** The nucleosome occupancies over SRATs are plotted, separated according to the positioning of SRAT start sites from the 3' end of genes. Color scheme is defined in C. **E.** The H3 K4 trimethylation ChIP-Seq over SRATs are plotted, separated according to the positioning of SRAT start sites from the 3' end of genes. Color scheme is defined in C.

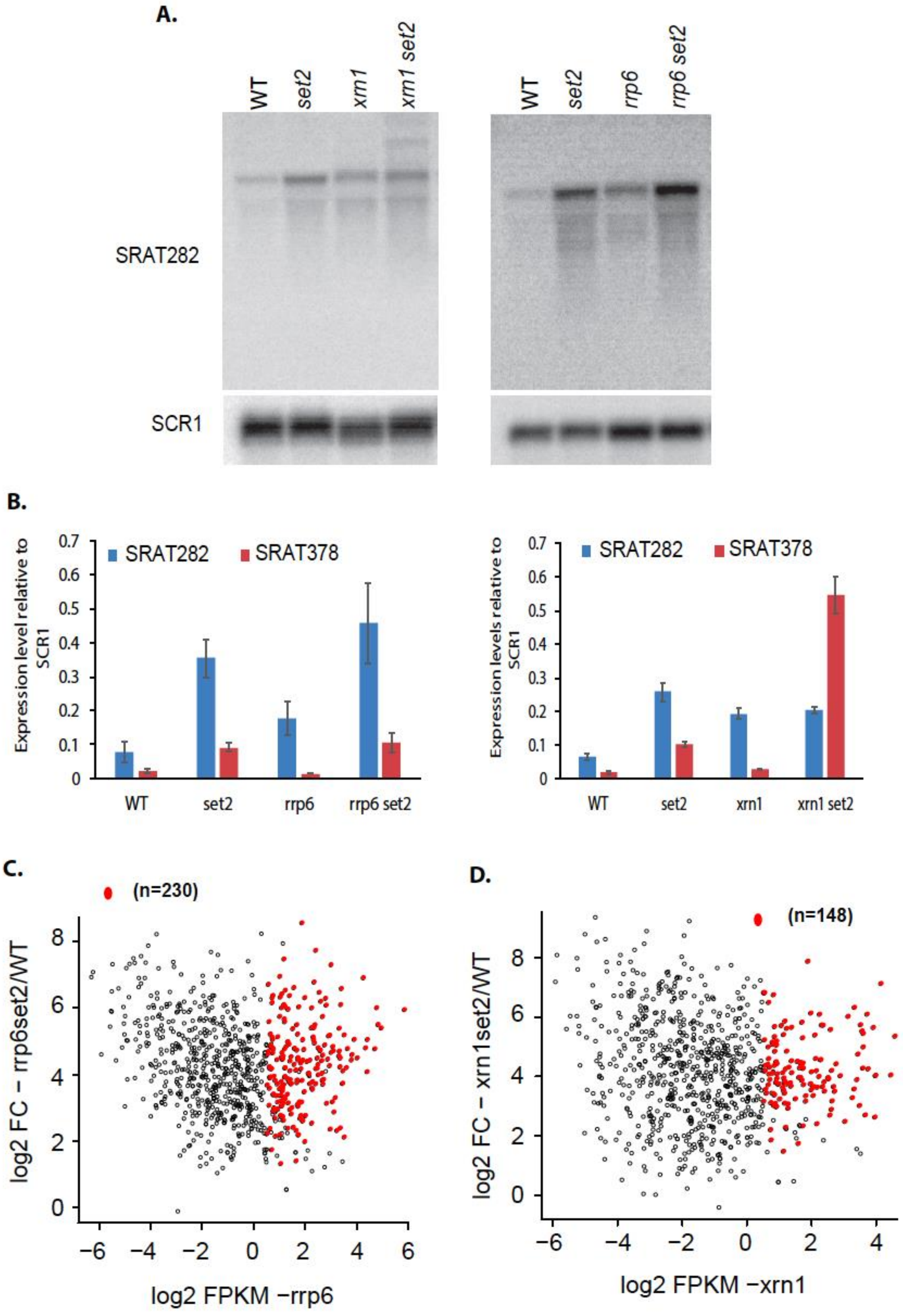

Supplementary Figure. 6: SRATs are differentially affected by the loss of RNA degradation machinery components. A. (Left) Strand specific northern blot probing for SRAT378 using total RNA in either the wild-type (WT-BY474), *SET2* deletion mutant (*set2*), *RRP6* deletion mutant (*rrp6*) and the *RRP6 SET2* double deletion mutant (*rrp6set2*). (Right)

Strand specific northern blot probing for SRAT378 using total RNA in either the wild-type (WT-BY474), *SET2* deletion mutant (*set2*), *XRN1* deletion mutant (*xrn1*) and the *XRN1 SET2* double deletion mutant (*xrn1set2*). *SCR1* is used as a loading control. **B.** Quantitation of strand specific northern blots indicating the expression level of selected SRATs, normalized to the level of *SCR1* in total RNA in the indicated mutants. Error bars denote the s.e.m of three independent repeats. **C.- D.** Scatter plots comparing the strand specific RNA-Seq transcript abundance of SRAT in the selected strain compared to the fold change in SRAT expression in the indicated mutant strains. The fold change of RNA abundance in indicated mutants with respect to the wild-type are distributed on the y-axis, while RNA abundance of the *RRP6* (*rrp6*) (C) or *XRN1* deletion (*xrn1*) (D) are distributed on the x-axis. The red dots indicate the SRATs that are significantly up-regulated in the mutant on the x-axis. The numbers are indicated on the top.

A.

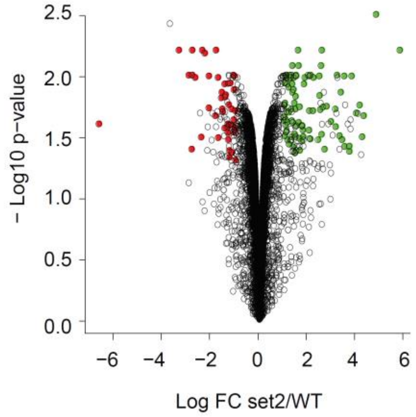

B.

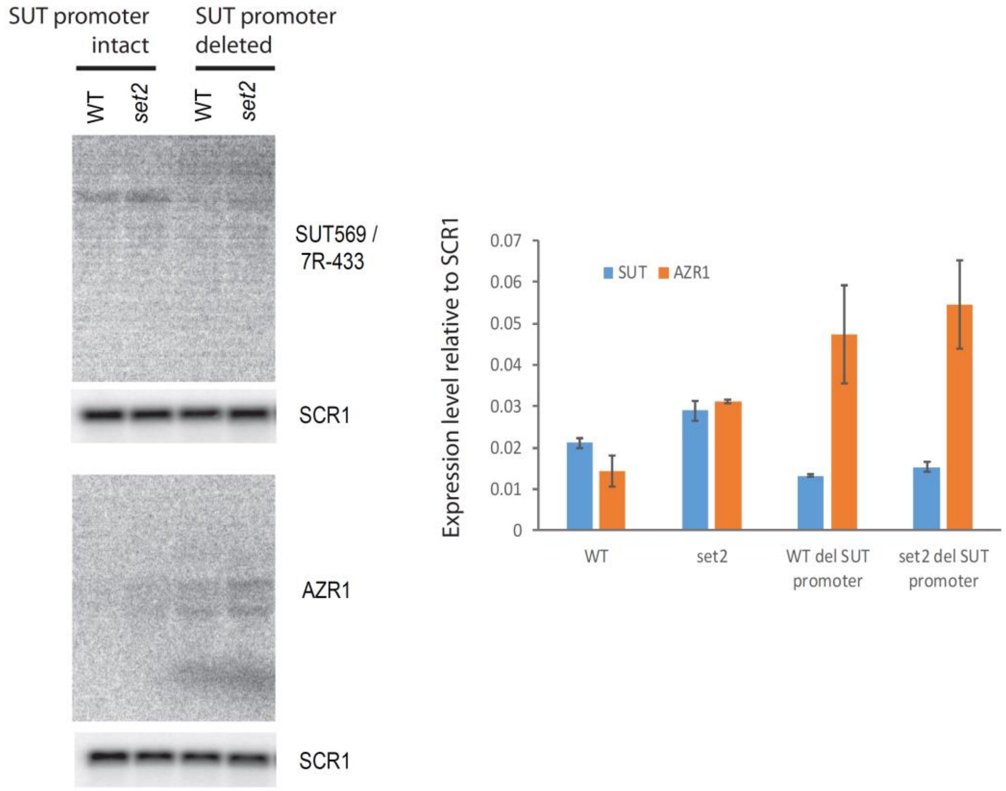

Supplementary Figure. 7: Loss of Set2 results in differential gene expression of selected protein coding genes. A. Volcano plot of the sense protein coding genes with the fold change in gene expression in the *set2* mutant over the wildtype in the x-axis versus the log transformed p-values on the y axis. Genes that have an RPKM of 5 or above and a 2-fold increase (green) or decrease (red) in expression are highlighted. **B.** (Left) Strand specific northern blot probing for either SUT569/7R-433 or *AZR1* using total RNA from either a strain with an intact SUT promoter or one with the SUT promoter deleted with a wild-type copy of Set2 (*SET2*) or *SET2* deletion mutant (*set2*). *SCR1* was used as a loading control. (Right) Quantitation of strand specific northern blots indicating the expression

level of selected SRATs, normalized to the level of *SCR1* in total RNA in the indicated mutants. Error bars denote the s.e.m of three independent biological repeats.

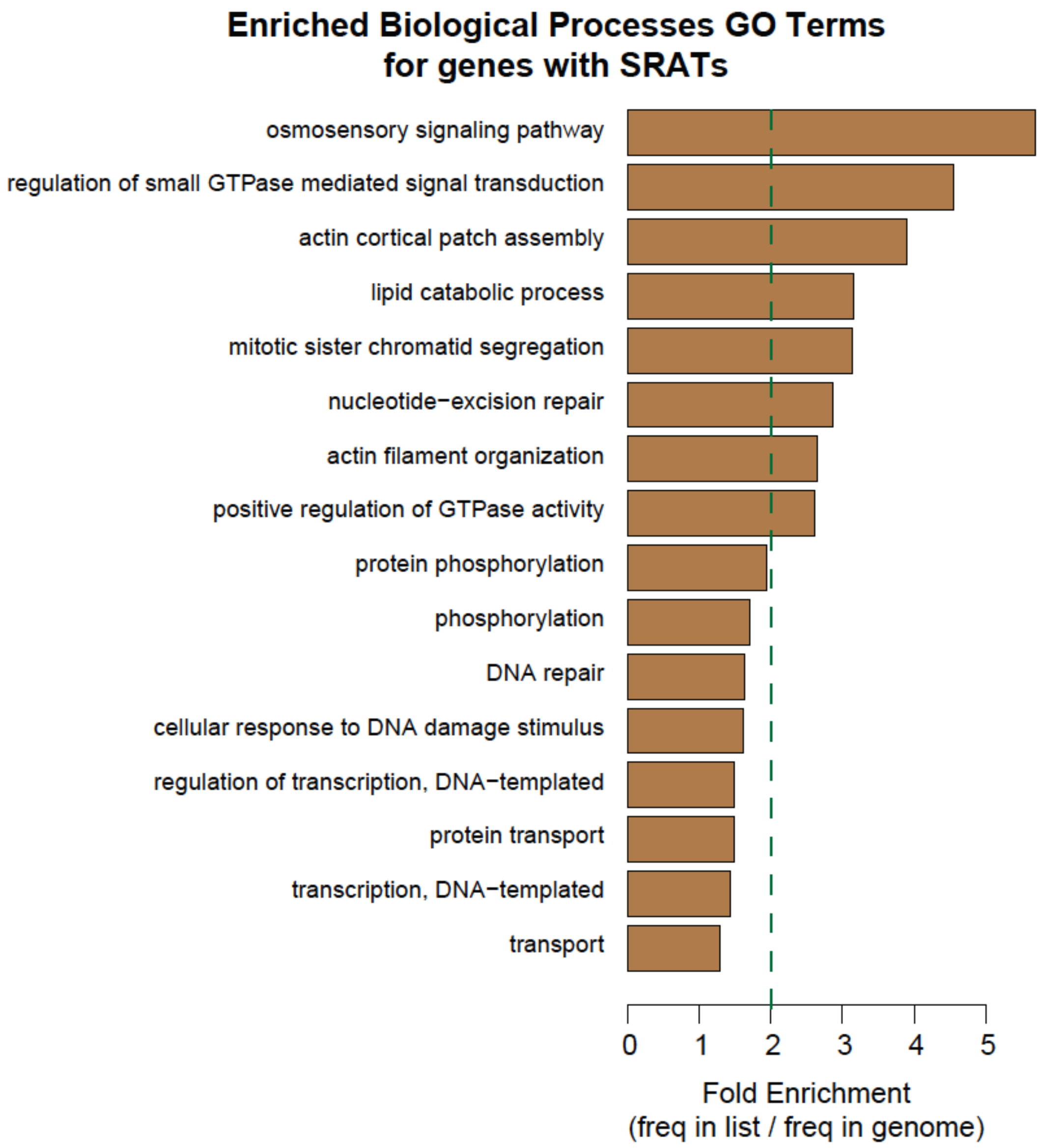

Supplementary Figure. 8: GO term enrichments genes with SRATs. Protein coding genes with embedded SRATs are enriched with GO terms for biological processes involved in the regulation of various stress conditions. The green line demarcates the terms that are enriched greater than 2-fold to the right.

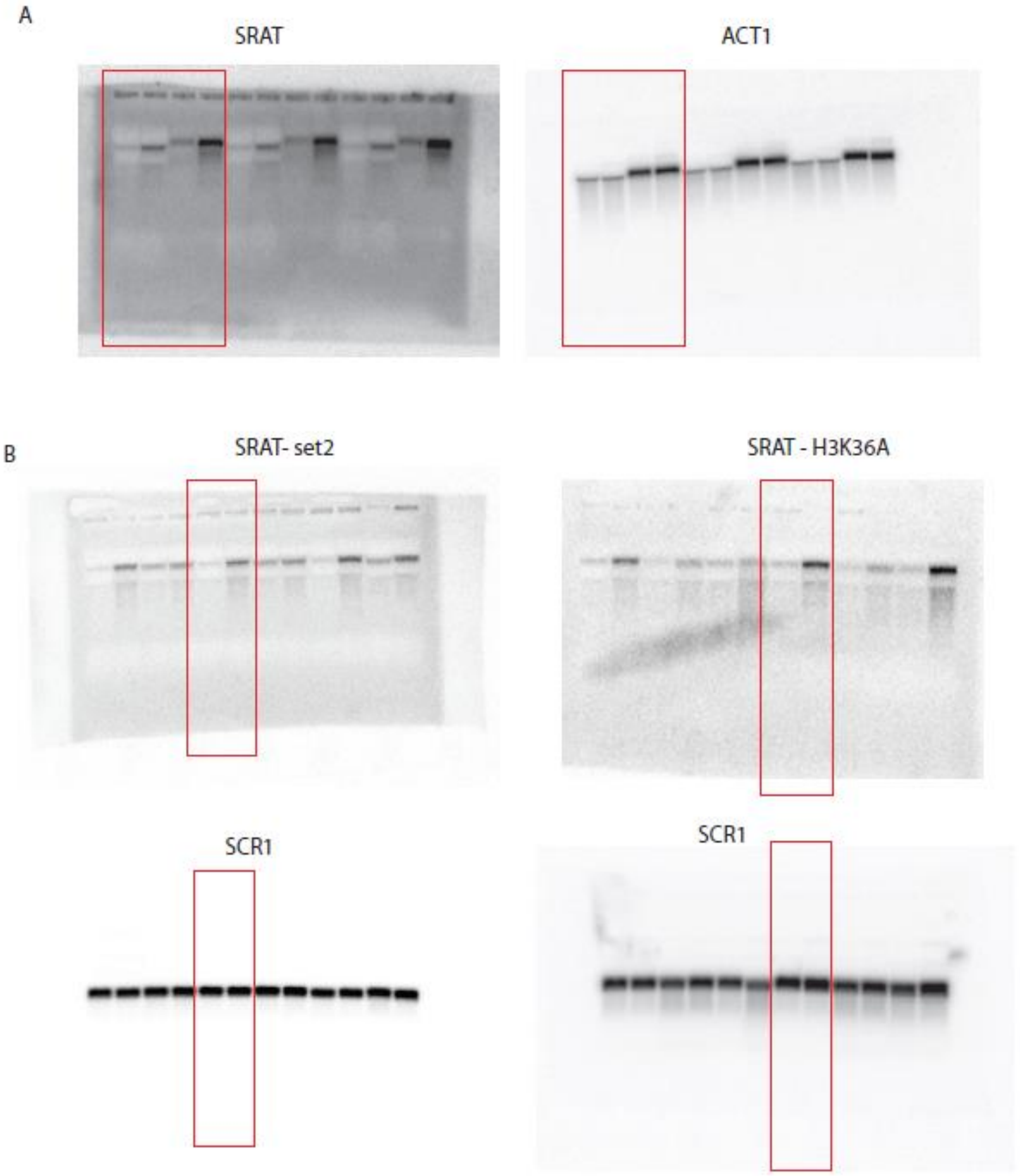
A
SRAT
ACT1
B
SRAT- set2
SRAT - H3K36A
SCR1
SCR1

C.

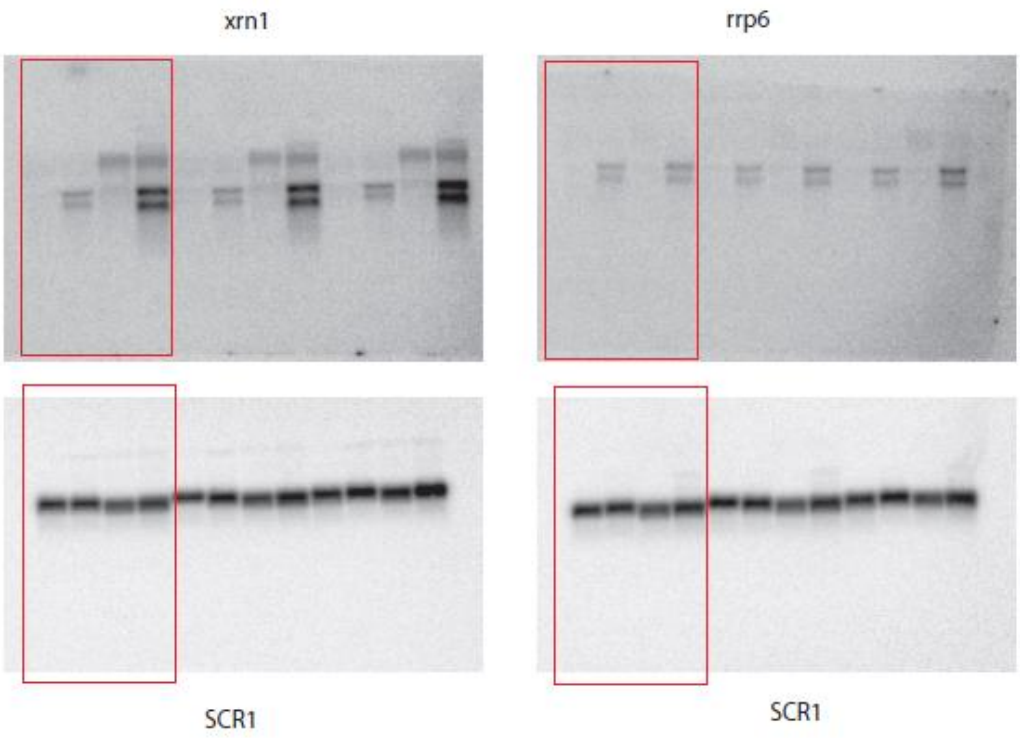

D.

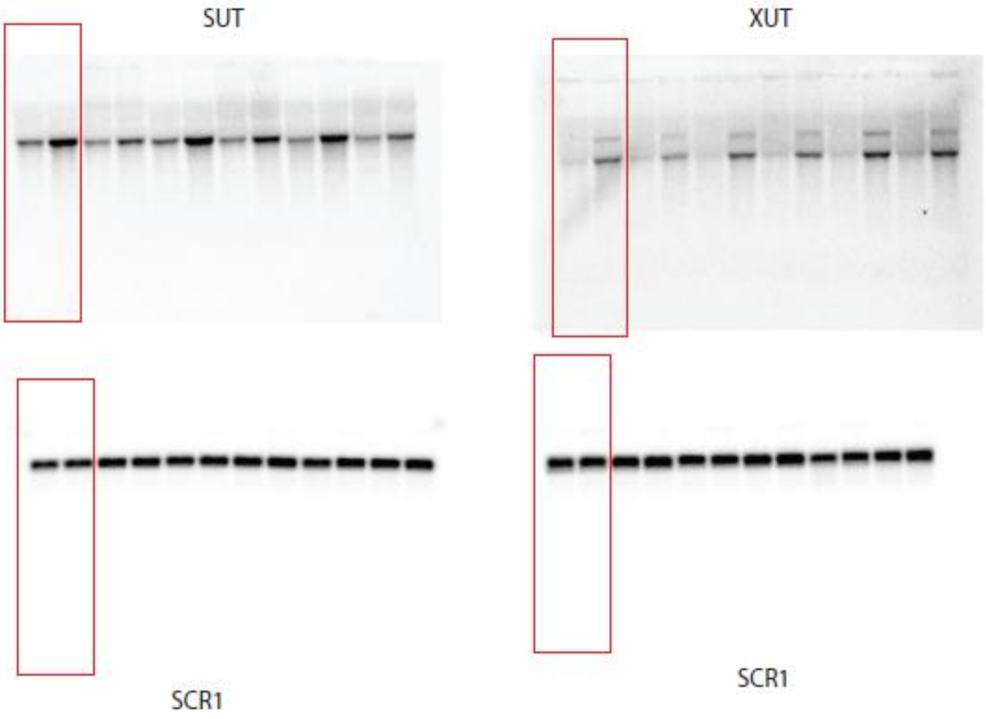

E.

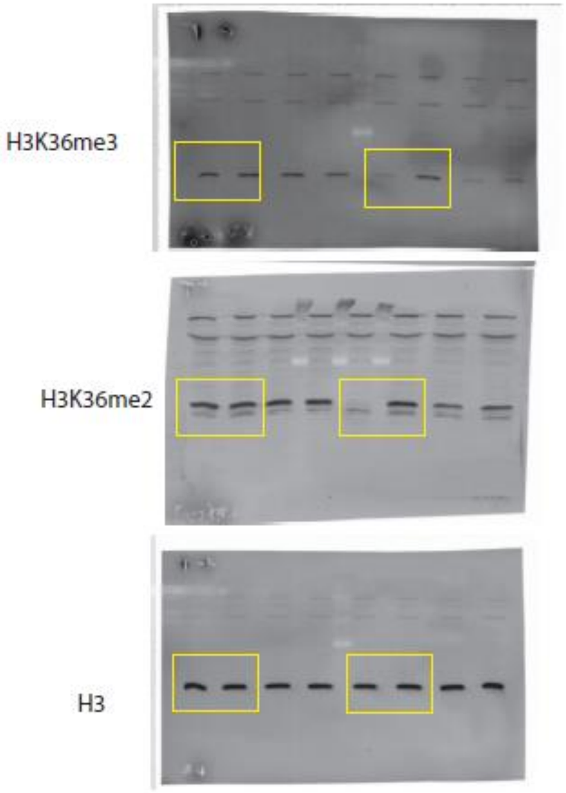

F.

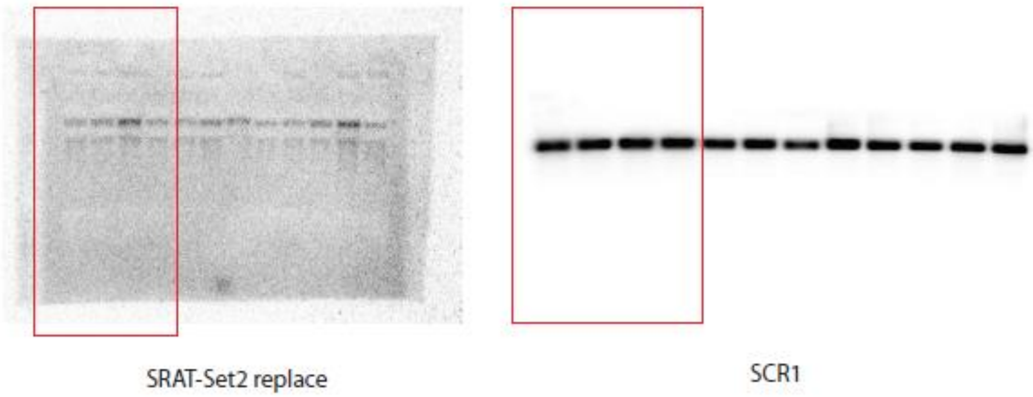

G.

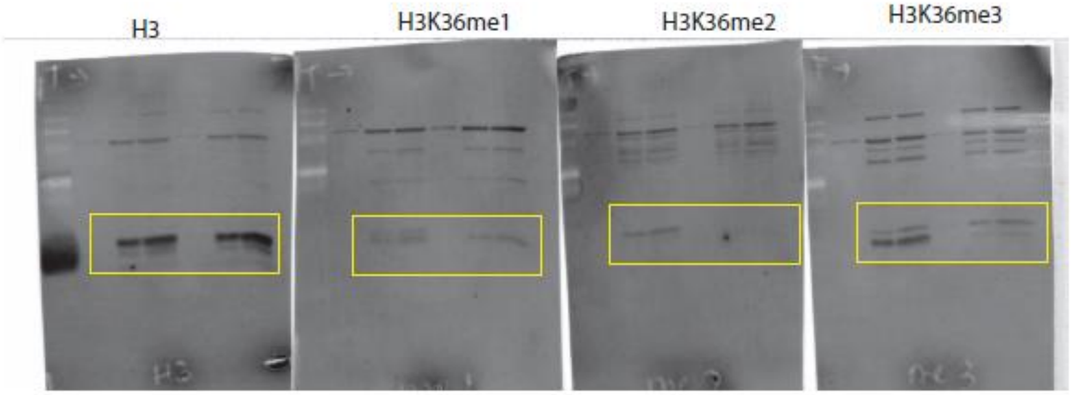
H3
H3K36me1
H3K36me2
H3K36me3

H.

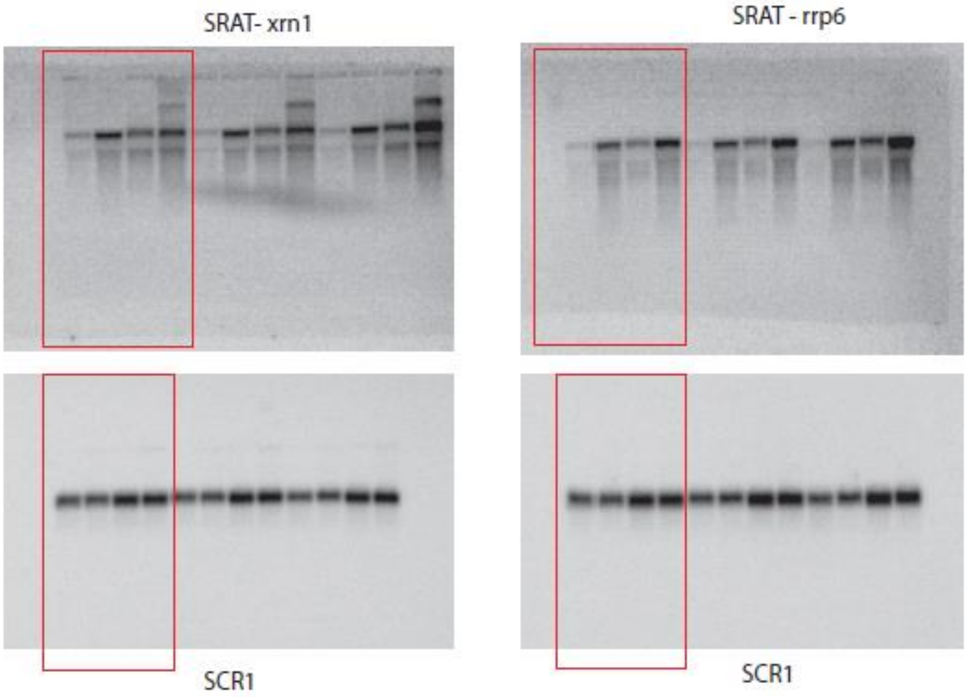
SRAT- xrn1
SRAT - rrp6
SCR1
SCR1

I.

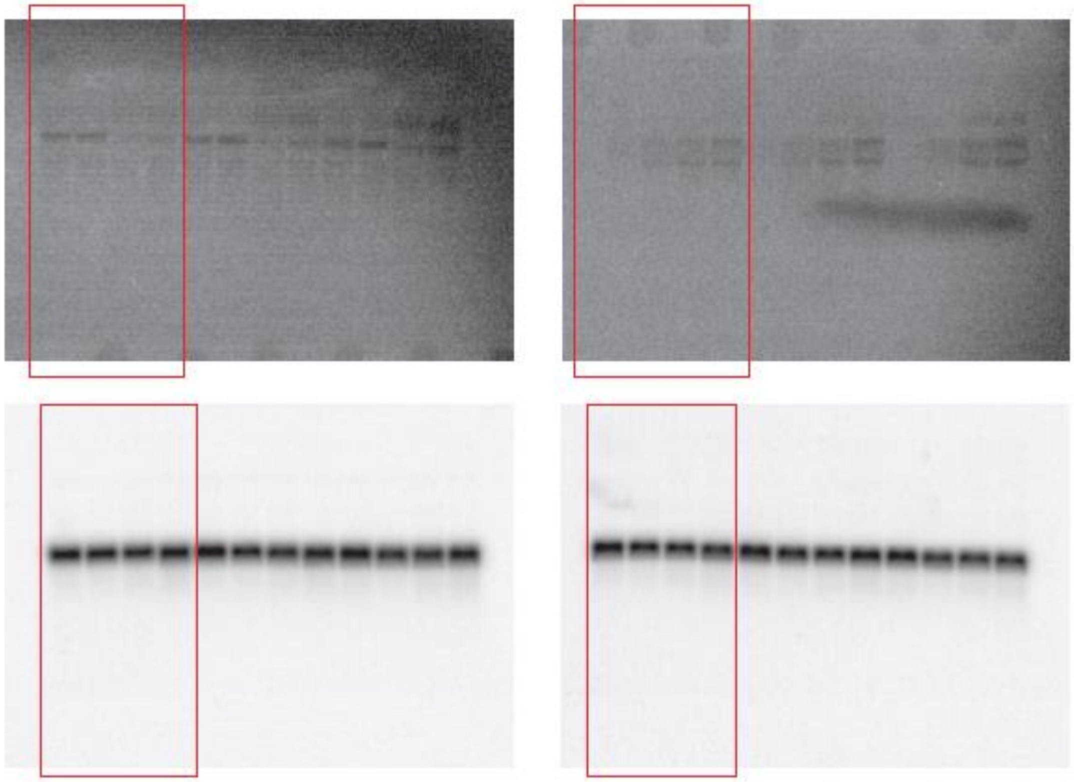

Supplementary Figure 9: Original figures used for representative blots and quantitation. A. Original and full blot for Fig. 1C. **B.** Original and full blot for Fig. 2C. **C.** Original and full blot for Fig. 5A. **D.** Original and full blot for Fig. 6C. **E.** Original and full blot for Supplementary Fig. 2C. **F.** Original and full blot for Supplementary Fig. 3E. **G.** Original and full blot for Supplementary Fig. 4A. **H.** Original and full blot for Supplementary Fig. 6A. **I.** Original and full blot for Supplementary Fig. 7B.
